# Feature tuning improves MAXENT predictions of the potential distribution of *Pedicularis longiflora* Rudolph and its variant

**DOI:** 10.7717/peerj.13337

**Published:** 2022-05-03

**Authors:** Ru Bao, Xiaolong Li, Jianghua Zheng

**Affiliations:** 1 College of Geographical Sciences, Xinjiang University, Urumqi, China; 2 Key Laboratory of Oasis Ecology of Ministry of Education, Xinjiang University, Urumqi, China; 3 College of Vocational and Technical, Xinjiang Teacher’s College (Xinjiang Education Institute), Urumqi, China; 4 Department of Natural Resources of Xinjiang Uygur Autonomous Region, Urumqi, China

**Keywords:** *Pedicularis longiflora* Rudolph, *Pedicularis longiflora* var. *tubiformis* (Klotzsch) Tsoong, MAXENT, Niche model, Potential distribution, Alpine medicinal plant

## Abstract

*Pedicularis longiflora* Rudolph and its variant (*P. longiflora* var. *tubiformis* (Klotzsch) Tsoong) are alpine plants and traditional Chinese medicines with important medicinal value, and future climate changes may have an adverse impact on their geographic distribution. The maximum entropy (MAXENT) model has the outstanding ability to predict the potential distribution region of species under climate change. Therefore, given the importance of the parameter settings of feature classes (FCs) and the regularization multiplier (RM) of the MAXENT model and the importance of add indicators to evaluate model performance, we used ENMeval to improve the MAXENT niche model and conducted an in-depth study on the potential distributions of these two alpine medicinal plants. We adjusted the parameters of FC and RM in the MAXENT model, evaluated the adjusted MAXENT model using six indicators, determined the most important ecogeographical factors (EGFs) that affect the potential distributions of these plants, and compared their current potential distributions between the adjusted model and the default model. The adjusted model performed better; thus, we used the improved MAXENT model to predict their future potential distributions. The model predicted that *P. longiflora* Rudolph and its variant (*P. longiflora* var. *tubiformis* (Klotzsch) Tsoong) would move northward and showed a decrease in extent under future climate scenarios. This result is important to predict their potential distribution regions under changing climate scenarios to develop effective long-term resource conservation and management plans for these species.

## Introduction

*Pedicularis longiflora* Rudolph and its variant (*P. longiflora* var. *tubiformis* (Klotzsch) Tsoong) belong to the series Longiflorae of the genus *Pedicularis* within the Orobanchaceae family (Olmstead et al., 2001; The Angiosperm Phylogeny Group, 2003). *P. longiflora* Rudolph is widely distributed in Gansu, Qinghai, Sichuan, the Tibet Autonomous Region and Yunnan in China, in alpine meadows and along streams, springs and seeps at altitudes of 2,100–5,300 m above sea level (http://www.iplant.cn/frps). It is used as a traditional medicine by Amchis in the Tibet Autonomous Region (China) to heal hepatic and pancreatic diseases (Angmo, Adhikari & Rawat, 2012). Moreover, its phytochemicals have antioxidant and antidiabetic functions (Yatoo et al., 2016, 2017). *P. longiflora* var. *tubiformis* (Klotzsch) Tsoong is widely distributed in the Tibet Autonomous Region, Yunnan, Sichuan, Qinghai and Gansu in China (Chen et al., 2013; Zhang et al., 2014), in alpine meadows and along streams, springs and seeps at altitudes of 2,700–5,300 m above sea level (http://www.iplant.cn/frps). The entire plant is an important traditional Tibetan medicine (Chinese name: Banchunmaxianhao (BCM)) (Yang, 1991; Chen et al., 2013). According to records on Qinghai economic plants, this plant has a good therapeutic effect on edema, hepatitis, spermatorrhea, cholecystitis and other diseases (Guo, 1987; Lan et al., 2018). Compared with *P. longiflora* Rudolph, the flowers of its variant (*P. longiflora* var. *tubiformis* (Klotzsch) Tsoong) are generally smaller, and there are two brown–red spots on the lower lip near the throat. In recent decades, global climate change (IPCC, 2014) has had a negative impact on the suitable habitat and even the survival of some species, especially the most precious natural medicinal resources (Shrestha, 2012; Yan et al., 2017). *P. longiflora* Rudolph and its variant (*P. longiflora* var. *tubiformis* (Klotzsch) Tsoong) are alpine plants and traditional Chinese medicines with important medicinal value, and future climate changes may have an adverse impact on their geographic distribution. Therefore, it is important to predict their potential distribution regions under changing climate scenarios to develop effective long-term resource conservation and management plans for these species.

Ecological niche models (ENMs, also referred to as species distribution models, SDMs (Elith & Leathwick, 2009; Miller, 2010)) have become an effective tool to predict the potential distribution and suitable habitat of target species (Akhter et al., 2017) and can be used in biogeography (Chen, Yang & Sun, 2011), conservation biology (Brambilla et al., 2013; Bernardes et al., 2013), landscape ecology (Amici et al., 2015), plant ecology (Gelviz-Gelvez et al., 2015) and restoration ecology (Fernández & Morales, 2016; Morales, Fernández & Baca-González, 2017). The maximum entropy (MAXENT) model is not only one of the most popular and widely used methods in ENMs (Merow, Smith & Silander, 2013) but also one of the best-performing techniques for dealing with presence-only data (Mateo et al., 2010; Sevestre & Benn, 2015). Although the current default settings in MAXENT are based on extensive empirical tuning research (Phillips & Dudík, 2008), recent studies have shown that they can result in poor model performance (Shcheglovitova & Anderson, 2013; Radosavljevic & Anderson, 2014). In addition, because artificial spatial autocorrelation between training and test data partitions (for example, sampling bias) can exaggerate the metrics used to evaluate model performance (Veloz, 2009; Wenger & Olden, 2012; Radosavljevic & Anderson, 2014), it may be necessary to add indicators to evaluate model performance.

For the above reasons, we selected the R package (ENMeval) to improve the predictive ability of ENMs; ENMeval facilitates the construction and evaluation of ENMs by improving the MAXENT model (Muscarella et al., 2014). In this study, we applied the improved MAXENT niche model to *P. longiflora* Rudolph and its variant (*P. longiflora* var. *tubiformis* (Klotzsch) Tsoong) with the goals of (a) comparing their potential distributions of the adjusted model and the default model under current climate scenarios, (b) determining the most important ecogeographical factors (EGFs) that affect the potential distributions of these plants, and (c) using the improved MAXENT model to predict their potential distributions under future climate scenarios.

## Materials and Methods

### Study region and species data collection

**Figure 1:**
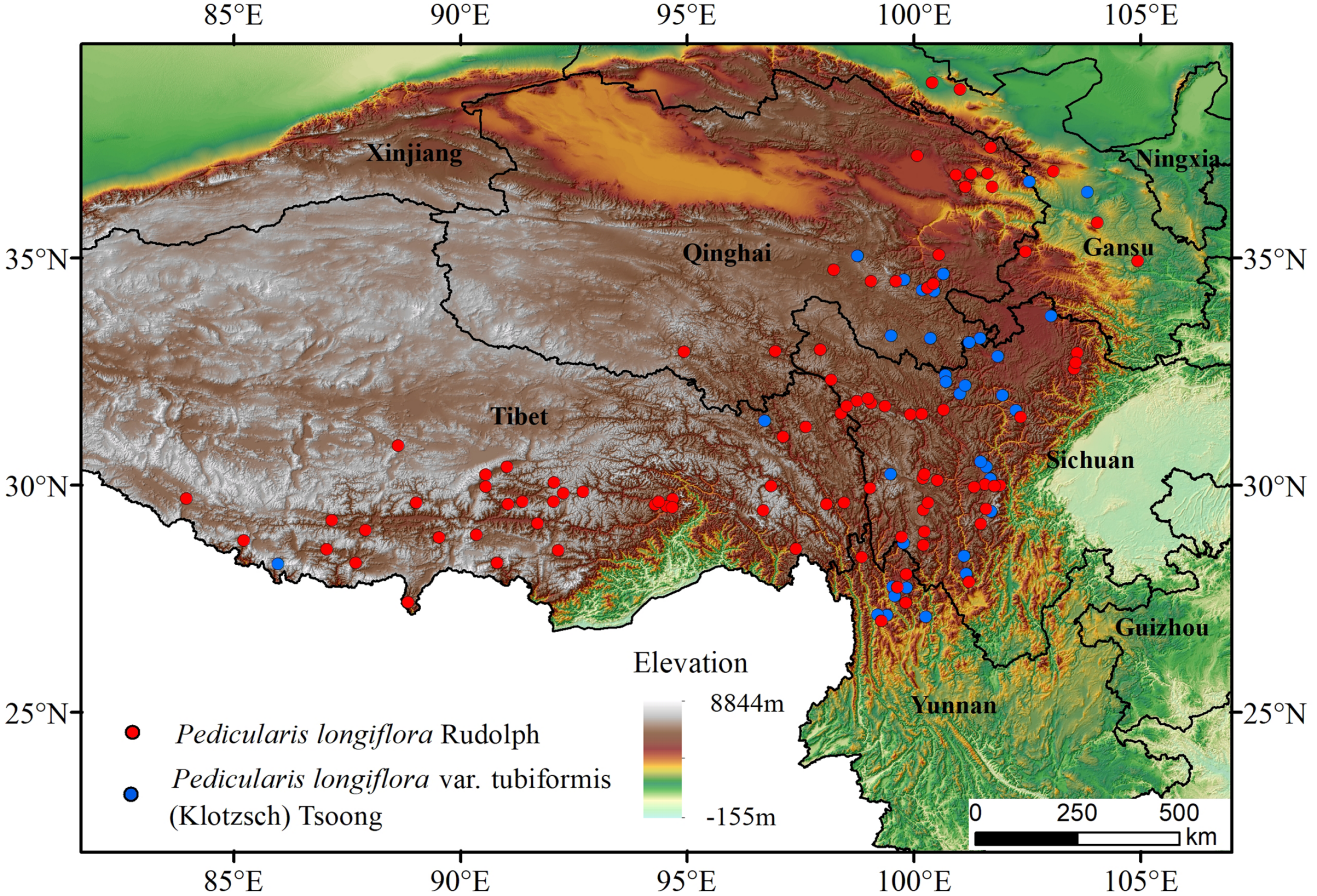
Species distribution occurrence records of *Pedicularis longiflora* Rudolph and *Pedicularis longiflora* var. *tubiformis* (Klotzsch) Tsoong in China and the Tibet Autonomous Region.

*P. longiflora* Rudolph is distributed mainly in China, and a small population is distributed in Mongolia, Russia, Nepal, Pakistan and India. Its variant (*P. longiflora* var. *tubiformis* (Klotzsch) Tsoong) is also distributed mainly in China, and a small population is distributed in Nepal and India. Our study region was China. After consulting the literature and collecting, comparing and analyzing data, we ultimately chose to use occurrence data downloaded from the Global Biodiversity Information Facility data portal (https://www.gbif.org/) (Xu et al., 2013). To improve the accuracy of prediction, we processed the occurrence data as follows: first, only points with accurate geographic location information were selected; second, duplicate points were deleted; finally, overdense points were deleted, and only one point was retained for each grid (1*1 km) (Zhang et al., 2020; von Takach et al., 2020). After the above steps, 103 *P. longiflora* Rudolph and 43 *P. longiflora* var. *tubiformis* (Klotzsch) Tsoong occurrence records were used for modeling and analysis (Deb et al., 2017) (Fig. 1).

### EGFs selection

To construct models for *P. longiflora* Rudolph and *P. longiflora* var. *tubiformis* (Klotzsch) Tsoong, we first considered a series of 22 EGFs related to their distributions (Table S1). These EGFs included 19 bioclimatic factors and three topographical factors. The bioclimatic data were downloaded from the Global Climate Data (WorldClim) datasets (http://www.worldclim.org) (Hijmans et al., 2005), and the topographical data were acquired from the Topobathy Dataset of the U.S. Geological Survey (USGS) (https://topotools.cr.usgs.gov/). All EGFs were resampled to a resolution of 30 arc s (ca. 1 km^2^). To minimize collinearity among the EGFs, we calculated the Pearson correlation coefficient and chose only the EGFs for which |*r*| ≤ 0.70 (*r* is the correlation coefficient) (Bosso et al., 2016; Delgado-Jaramillo et al., 2020). Although both *P. longiflora* Rudolph and *P. longiflora* var. *tubiformis* (Klotzsch) Tsoong belong to the series Longiflorae in the *Pedicularis* genus, their numbers of sample points and distributions are different, indicating the existence of habitat heterogeneity. Therefore, the eight EGFs (Tables 1 and 2) that were selected to construct models through EGF selection were not exactly the same.

**Table 1:** Ecogeographical factors (EGFs) applied to predict the potential distribution of *Pedicularis longiflora* Rudolph.

Type	Ecogeographical factors (EGFs)	Units
Topo-graphical	Aspect	°
	Slope	°
Bio-climatic	Annual Mean Temperature	°C
	Mean Diurnal Range (Mean of monthly (max temp−min temp))	°C
	Temperature Seasonality (standard deviation *100)	C of V
	Annual Precipitation	mm
	Precipitation Seasonality (Coefficient of Variation)	C of V
	Precipitation of Coldest Quarter	mm

**Table 2:** Ecogeographical factors (EGFs) applied to predict the potential distribution of *Pedicularis longiflora* var. *tubiformis* (Klotzsch) Tsoong.

Type	Ecogeographical factors (EGFs)	Units
Topo-graphical	Aspect	°
	Slope	°
Bio-climatic	Annual Mean Temperature	°C
	Mean Diurnal Range (Mean of monthly (max temp−min temp))	°C
	Isothermality (Mean Diurnal Range/Temperature Annual Range) (*100)	–
	Annual Precipitation	mm
	Precipitation Seasonality (Coefficient of Variation)	C of V
	Precipitation of Coldest Quarter	mm

### Model tuning

Our parameter tuning to the MAXENT model was achieved mainly by applying the ENMeval software package to adjust the parameters of FC and RM. We referred to recent studies on parameter adjustment for species, including adjusting regularization parameters and setting feature types (*i.e*., linear, quadratic, product, threshold, and hinge) (Anderson & Gonzalez, 2011; Dilts et al., 2019). After using the Checkerboard 2 method to divide the occurrence data, we constructed the models with RM values ranging from 1 to 5 (in increments of one) and with six different FC combinations (H, L, LQ, LQH, LQHP, LQHPT; where H = hinge, L = linear, Q = quadratic, P = product, and T = threshold) (Dilts et al., 2019). This approach resulted in 60 individual model runs.

### Model evaluation

Our evaluation of the improved MAXENT model was achieved mainly by using multiple indicators provided by ENMeval. The specific indicators are as follows: (a) the area under the curve (AUC_TEST_) of the receiver operating characteristic curve based on the test data is calculated. This indicator can measure the model’s ability to distinguish the hidden occurrence location conditions from the background sample conditions; (b) the difference between the training and test AUC (AUC_DIFF_) is calculated. This indicator can quantify overfitting, and the value of overfitting is expected to be very high (Warren & Seifert, 2011); (c) two omission rates are calculated. These omission rates are compared with their theoretically expected omission rates to quantify the model overfitting: the ratio of the test localities to MAXENT’s output value lower than that corresponding to (i) the training locality with the lowest value (*i.e*., the minimum training presence, MTP = 0% training omission) or (ii) a 10% omission rate for the training localities (= 10% training omission) (Pearson et al., 2007); (d) the mean and variance of each of the above four indicators are calculated (corrected for the non-independence of the k iterations, (Shcheglovitova & Anderson, 2013)); and (e) the sample size correction Akaike information criterion (AICc) value, ΔAICc and AICc weights of each complete model are calculated, providing information about the relative quality of the model for the given data (Burnham & Anderson, 2004; Warren & Seifert, 2011). Because AICc is calculated using the complete dataset, it is not affected by the method selected for data partitioning.

### Model application

We used CCSM4 global climate model downloaded from Global Climate Data (WorldClim) datasets (http://www.worldclim.org) (Hijmans et al., 2005) and the improved MAXENT model to predict and analyze future potential distributions of *P. longiflora* Rudolph and *P. longiflora* var. *tubiformis* (Klotzsch) Tsoong. We applied CCSM4 to two time series, namely, 2050 (average for 2041–2060) and 2070 (average for 2061–2080), with three representative concentration pathways (RCPs), namely, RCP2.6, RCP6.0 and RCP8.5. We used MaxEnt version 3.4.0 (http://biodiversityinformatics.amnh.org/open_source/maxent/) (Phillips et al., 2017) to compute the possible suitable habitats of these plants with suitability ranging from 0 (lowest suitability) to 1 (highest suitability). We contrasted the area changes and the centroid shifts in their potential distribution regions between the current and every future climate scenario. According to expert experience and relevant literature, habitats were classified into four categories based on suitability: “high suitability” (0.6–1), “moderate suitability” (0.4–0.6), “low suitability” (0.2–0.4) and “unsuitable” (0–0.2) (Zhang et al., 2018).

## Results

### Model performance and comparison

**Figure 2:**
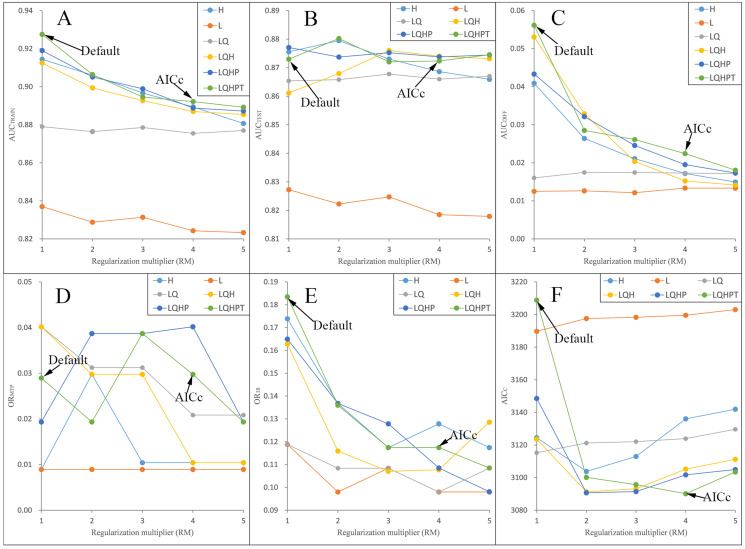
Evaluation indicators for *Pedicularis longiflora* Rudolph resulting from models constructed across a range of feature class (FC) combinations and regularization multiplier (RM) values. (FC) combinations (H, L, LQ, LQH, LQHP, LQHPT; where H = hinge, L = linear, Q = quadratic, P = product, and T = threshold) and (RM) values ranging from 1 to 5 (incremental one). (A) AUC_TRAIN_; (B) AUC_TEST_; (C) AUC_DIFF_; (D) OR_MTP_; (E) OR_10_; (F) AIC_C_.

**Figure 3:**
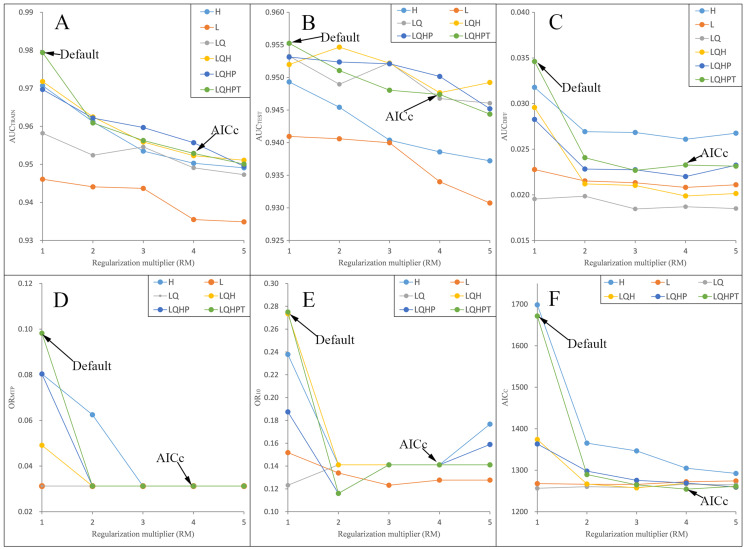
Evaluation indicators for *Pedicularis longiflora* var. *tubiformis* (Klotzsch) Tsoong resulting from models constructed across a range of feature class (FC) combinations and regularization multiplier (RM) values. (FC) combinations (H, L, LQ, LQH, LQHP, LQHPT; where H = hinge, L = linear, Q = quadratic, P = product, and T = threshold) and (RM) values ranging from 1 to 5 (incremental 1). (A) AUC_TRAIN_; (B) AUC_TEST_; (C) AUC_DIFF_; (D) OR_MTP_; (E) OR_10_; (F) AIC_C_.

We ran and compared the MAXENT models after and before adjusting the RM and FC parameters—namely, the adjusted model and the default model, respectively—on *P. longiflora* Rudolph and *P. longiflora* var. *tubiformis* (Klotzsch) Tsoong and obtained the following results: (a) the adjusted model performed better. We calculated and compared AICc, AUC_DIFF_, OR_MTP_ and OR_10_ of the adjusted model and the default model. According to the AIC, when RM = 4 and the FCs were L, Q, H, P and T, the AICc values of *P. longiflora* Rudolph and *P. longiflora* var. *tubiformis* (Klotzsch) Tsoong were the minimum values (Figs. 2 and 3); therefore, we chose this combination of parameters for the adjusted optimization model to predict their future potential distributions. The parameters of the default model were set as follows: RM = 1, FC = L, Q, H, P and T. Although we did not find a general trend for the FCs, the RM values of the models selected by AICc were higher than the default value of 1.0. Since higher regularization “smooths” the response curve by imposing higher penalties on the addition of parameters, it indicated that the default setting model tended to lead to more complex models compared to the models selected by AICc. In addition, the model selected by AICc also had lower AUC_DIFF_, OR_MTP_ and OR_10_ values than the default model, indicating that the model selected by AICc potentially had less overfitting. (b) The forecast effect of the adjusted model was better. For *P. longiflora* Rudolph and *P. longiflora* var. *tubiformis* (Klotzsch) Tsoong, the predictive map from the default model showed that there were large differences in the values of several adjacent pixels within the suitable regions that were not at the edges, that is, there were more isolated pixels inside the regions. However, this predictive map was not consistent with the actual known plant distribution. In contrast, the tuned model did not display this pattern as the values of adjacent pixels in the suitable regions were more continuous.

### The most important EGFs

**Figure 4:**
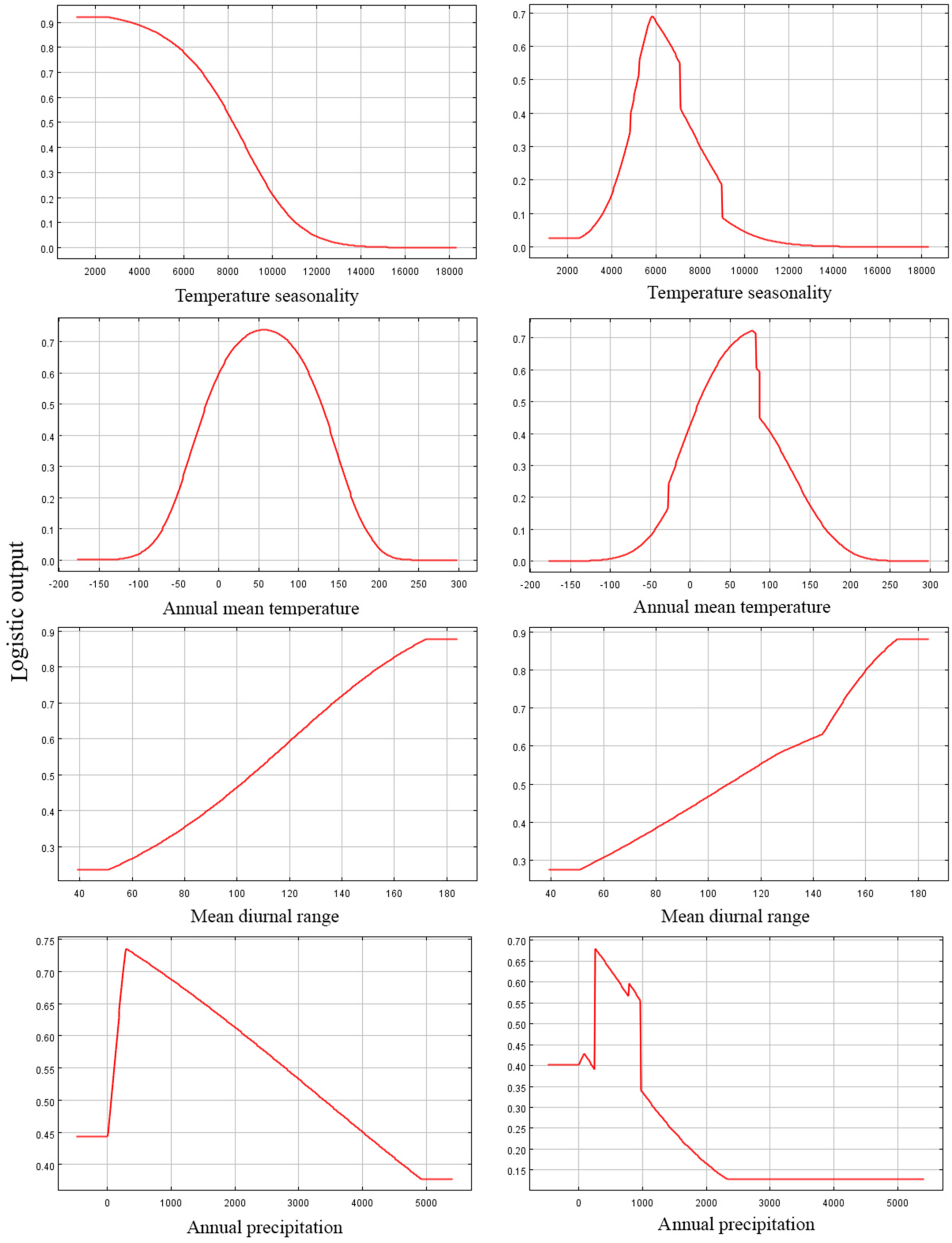
Response curves of four environmental variables in the current potential distribution model for *Pedicularis longiflora* Rudolph. Temperature seasonality; Annual mean temperature (°C); Mean diurnal range (°C); Annual precipitation (mm). X-axis – Values of predictive variables; Y-axis – Logistic probability of presence/suitability.

Among the eight EGFs used for modeling, in both the adjusted model and the default model, the most important EGFs that affected the potential distributions of *P. longiflora* Rudolph were temperature seasonality, annual mean temperature, mean diurnal range and annual precipitation. The contributions of the four important EGFs of the adjusted model were temperature seasonality (49.20%), annual mean temperature (36.00%), mean diurnal range (7.30%) and annual precipitation (5.70%), with a total contribution of 98.20%. The contributions of the same 4 EGFs in the default model were temperature seasonality (46.40%), annual mean temperature (34.60%), mean diurnal range (10.70%) and annual precipitation (4.30%), with a total contribution of 96.00%. These climatic factors demonstrated that *P. longiflora* Rudolph is a temperature-sensitive and precipitation-sensitive plant species and that climate is its dominant control. For the topographic conditions, 2 EGFs had low contributions. The contributions of aspect (1.40%) and slope (0.40%) accounted for only 2.80% of the total contribution in the adjusted model, and the contributions of aspect (1.70%) and slope (1.00%) accounted for only 2.70% of the total contribution in the default model. These results showed that topographic conditions had very limited influences on the spatial distribution of *P. longiflora* Rudolph. The jackknife test showed that temperature seasonality was the most useful environmental factor in both the adjusted model and the default model. According to the response curve of the leading environmental variables (Fig. 4), the most suitable habitat conditions for *P. longiflora* Rudolph were as follows: in the adjusted model, temperature seasonality was 3,701–8,546; annual mean temperature was −22 to 137 °C; mean diurnal range was 97–167 °C, and annual precipitation was 218–1,567 mm; in the default model, temperature seasonality was 4,263–8,990; annual mean temperature was −23 to 137 °C; mean diurnal range was 95–167 °C, and annual precipitation was 238–1,088 mm. *P. longiflora* Rudolph is thus suitable for distribution in cold and humid climates and has low temperature tolerance.

**Figure 5:**
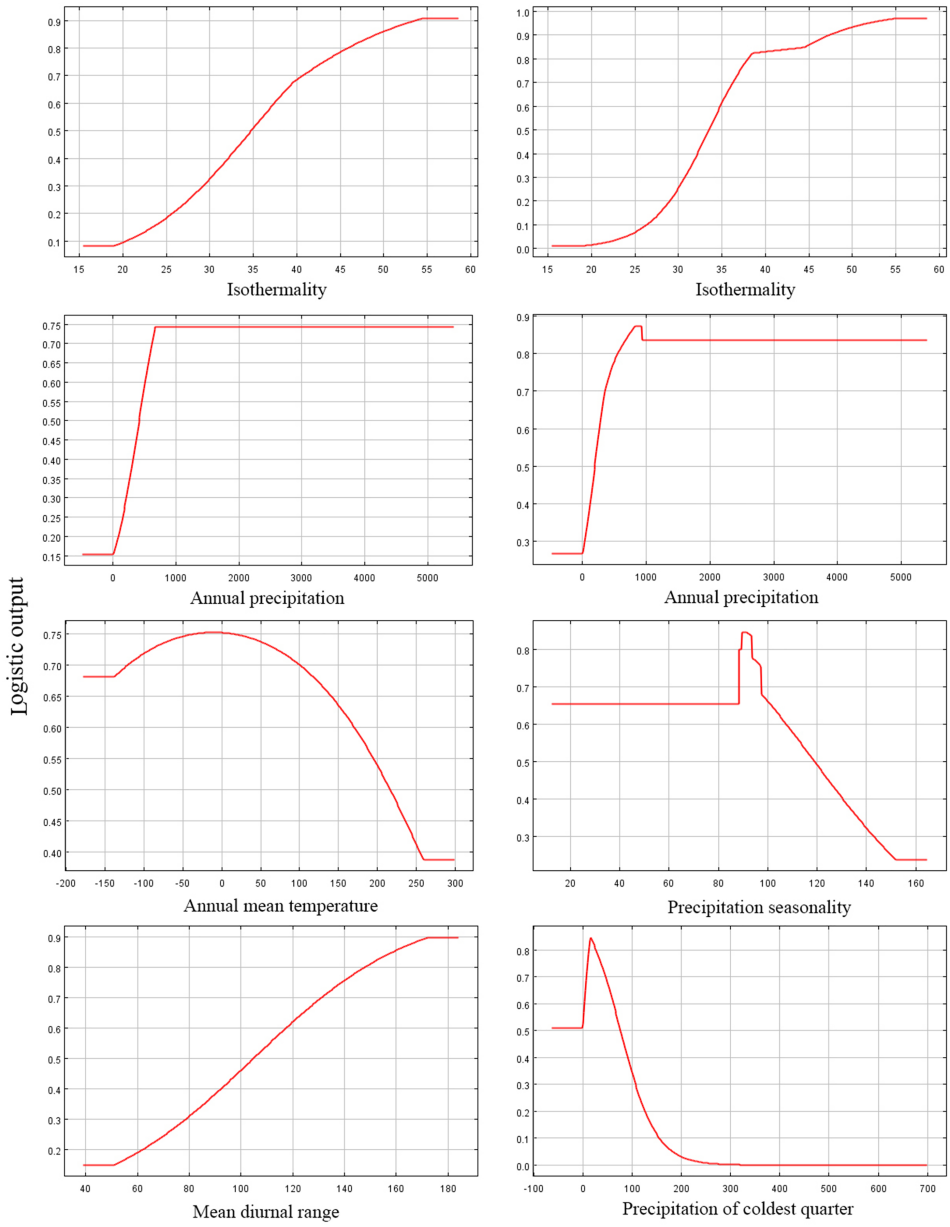
Response curves of six environmental variables in the current potential distribution model for *Pedicularis longiflora* var. *tubiformis* (Klotzsch) Tsoong. Isothermality (%); Annual precipitation (mm); Annual mean temperature (°C); Precipitation seasonality; Mean diurnal range (°C); Precipitation of coldest quarter (mm). X-axis – Values of predictive variables; Y-axis – Logistic probability of presence/suitability.

Among the eight EGFs used for modeling, in the adjusted model, the most important EGFs that affected the potential distribution of *P. longiflora* var. *tubiformis* (Klotzsch) Tsoong were isothermality, annual precipitation, annual mean temperature and mean diurnal range. The contributions of the four important EGFs were isothermality (61.50%), annual precipitation (21.20%), annual mean temperature (8.90%) and mean diurnal range (7.00%), with a total contribution of 98.60%. However, in the default model, the most important EGFs that affected the potential distribution of *P. longiflora* var. *tubiformis* (Klotzsch) Tsoong were isothermality, annual precipitation, precipitation seasonality and precipitation in the coldest quarter. The contributions of the four important EGFs were isothermality (55.90%), annual precipitation (14.60%), precipitation seasonality (10.10%) and precipitation in the coldest quarter (8.70%), with a total contribution of 95.20%. Similar to *P. longiflora* Rudolph, these climatic factors demonstrated that *P. longiflora* var. *tubiformis* (Klotzsch) Tsoong is also a precipitation-sensitive and temperature-sensitive plant species and that climate is its dominant niche factor. For the topographic conditions, two EGFs had low contributions. Slope and aspect had no contributions in the adjusted model, and the contributions of slope (2.30%) and aspect (0.50%) accounted for only 2.80% of the total contribution in the default model. These results showed that topographic conditions also had very limited influences on the spatial distribution of *P. longiflora* var. *tubiformis* (Klotzsch) Tsoong. The jackknife test showed that isothermality was the most useful environmental factor in both the adjusted model and the default model. According to the response curve of the leading environmental variables (Fig. 5), the most suitable habitat conditions for *P. longiflora* var. *tubiformis* (Klotzsch) Tsoong were as follows: in the adjusted model, isothermality was 38–53, annual precipitation was 327–1,645 mm, annual mean temperature was −82 to 222 °C, and mean diurnal range was 109–166 °C; in the default model, isothermality was 38–50, annual precipitation was 276–1,453 mm, precipitation seasonality was 65–116, and precipitation in the coldest quarter was 6–63 mm. Similar to *P. longiflora* Rudolph, *P. longiflora* var. *tubiformis* (Klotzsch) Tsoong is also suitable for distribution in humid and cold climates and has low temperature tolerance.

### Predictions of current potential distribution

**Figure 6:**
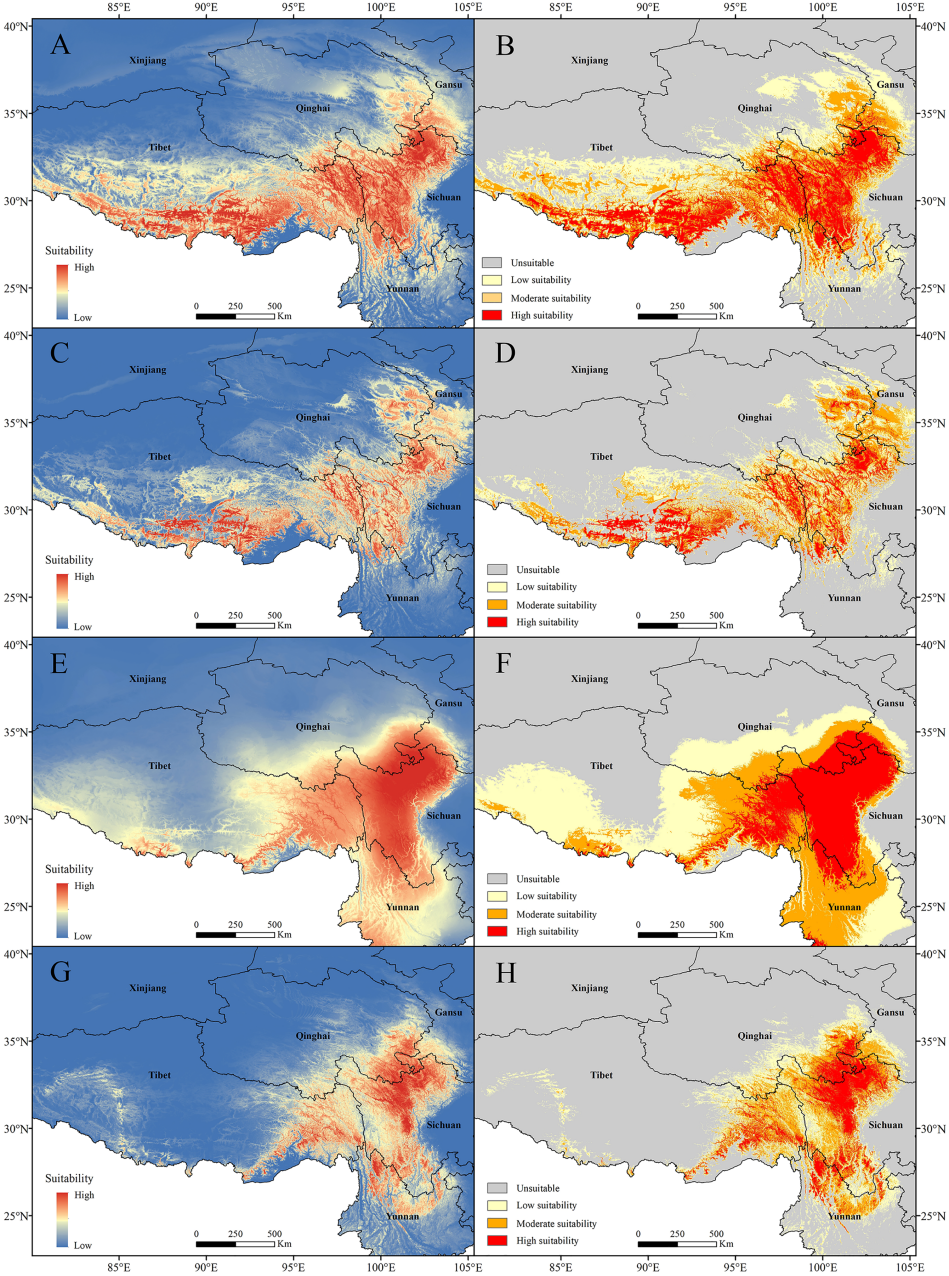
Models with the AICc-chosen setting (A), (B), (E), (F) and the default setting (C), (D), (G), (H) predicting current potential distributions (A), (C), (E), (G) and four categories suitability habitats (B), (D), (F), (H). (A)–(D) *Pedicularis longiflora* Rudolph; (E)–(H) *Pedicularis longiflora* var. *tubiformis* (Klotzsch) Tsoong.

The current potential distribution map for *P. longiflora* Rudolph in China is shown in Fig. 6. Our study showed that in the adjusted model, its high suitability area was 33.31 ha and was distributed mainly in western Sichuan and southern and eastern Tibet; the moderate suitability area was 44.86 ha and was distributed mainly in southwestern Gansu, eastern Qinghai, and southern and eastern Tibet; and the low suitability area was 61.21 ha and was distributed mainly in southwestern Gansu, eastern Qinghai and middle Tibet. In the default model, the high suitability area was 16.51 ha, the medium suitability area was 33.73 ha, and the low suitability area was 45.51 ha, and its main distribution regions were similar to those in the adjusted model. The area of the suitable regions predicted by the default model was smaller than that predicted by the adjusted model.

The current potential distribution map for *P. longiflora* var. *tubiformis* (Klotzsch) Tsoong in China is shown in Fig. 6. Our study showed that its high suitability area was 42.06 ha and was distributed mainly in southwestern Gansu, southeastern Qinghai, western Sichuan, eastern Tibet and northwestern Yunnan; the moderate suitability area was 53.23 ha and was distributed mainly in southwestern Gansu, southeastern Qinghai, eastern Tibet and western Yunnan; and the low suitability area was 74.17 ha and was distributed mainly in southwestern Gansu, southeastern Qinghai, eastern and southern Tibet and eastern Yunnan. In the default model, the high suitability area was 16.90 ha, the medium suitability area was 30.02 ha, the low suitability area was 37.10 ha, and its main distribution regions were similar to those of the adjusted model. The area of the suitable regions for *P. longiflora* var. *tubiformis* (Klotzsch) Tsoong predicted by the default model was also smaller than that predicted by the adjusted model.

### Predictions of the future potential distribution

For *P. longiflora* Rudolph, our study showed that compared with the current climate scenario, in the high suitability regions, the areas of future potential distribution regions were reduced. The lost suitable habitats were distributed mainly at the edges of the two large suitable regions; the newly added suitable habitats were located mainly in the middle of suitable regions, and the stable suitable habitats were also distributed mainly in the middle of suitable regions.

**Figure 7:**
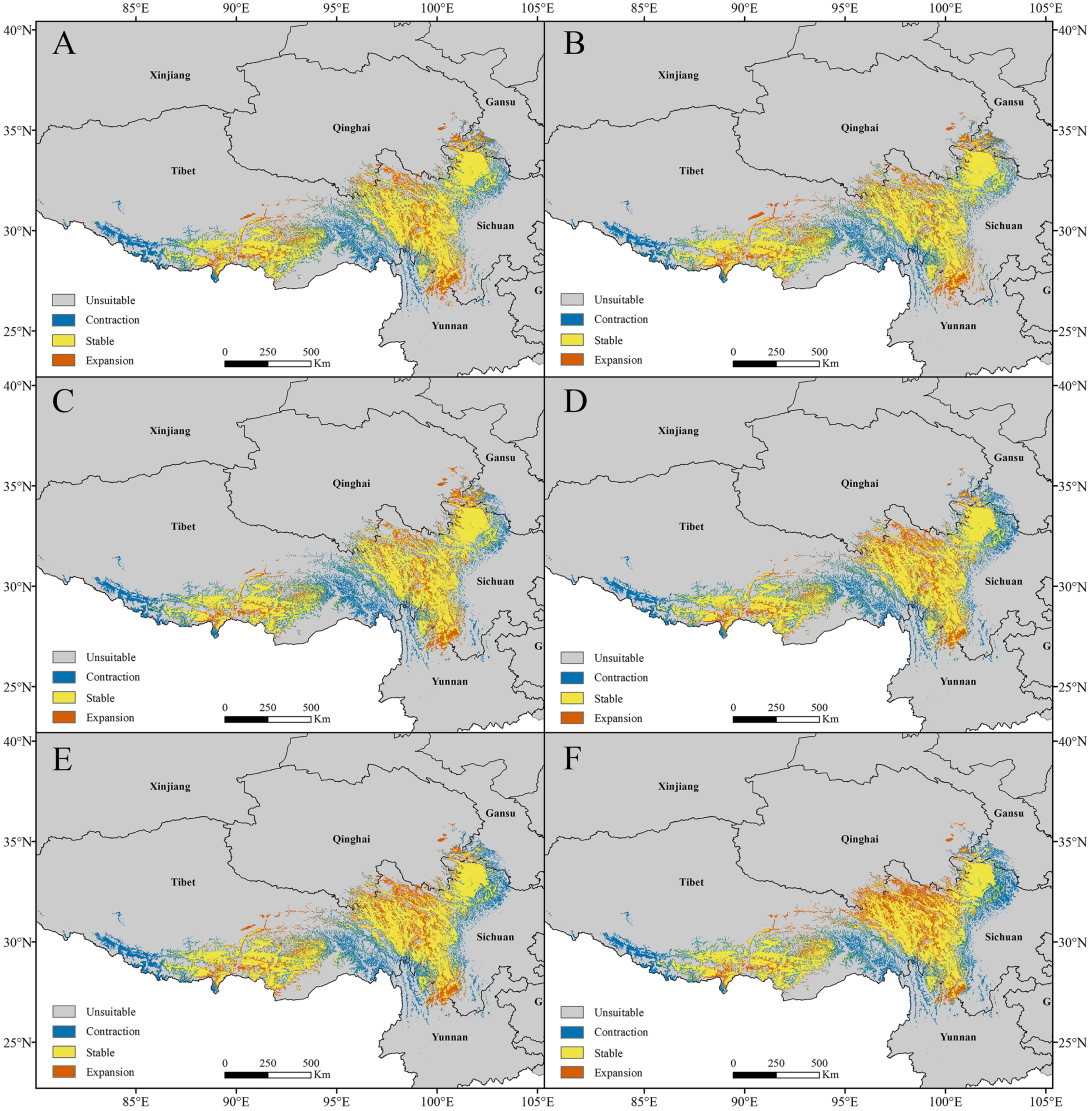
Changes in the potential distribution of *Pedicularis longiflora* Rudolph under climate change scenarios. (A) RCP2.6-2050; (B) RCP2.6-2070; (C) RCP6.0-2050; (D) RCP6.0-2070; (E) RCP8.5-2050; (F) RCP8.5-2070.

In the RCP2.6 scenarios, the model prediction showed that 20.30% of the highly suitable area would be lost in 2050 and that 19.33% would be lost in 2070. In the RCP6.0 scenarios, the model prediction showed that 21.64% of the highly suitable area would be lost in 2050 and that 20.84% would be lost in 2070. In the RCP8.5 scenarios, the model prediction showed that 21.14% of the highly suitable area would be lost in 2050 and that 20.94% would be lost in 2070 (Fig. 7). In general, the highly suitable area of the future potential distribution regions would be decreased by 4.08% in 2050 and 3.68% in 2070 under the RCP2.6 scenarios, by 6.77% in 2050 and 5.57% in 2070 under the RCP6.0 scenarios, and by 4.08% in 2050 and 1.51% in 2070 under the RCP8.5 scenarios.

For *P. longiflora* var. *tubiformis* (Klotzsch) Tsoong, our study showed that compared with the current climate scenario, in the high suitability regions, the highly suitable areas of future potential distribution regions were reduced. The lost suitable habitats were distributed mainly at the edges of suitable regions; the newly added suitable habitats were also mainly located at the edges of suitable regions, and the stable suitable habitats were distributed mainly in the middle of suitable regions.

**Figure 8:**
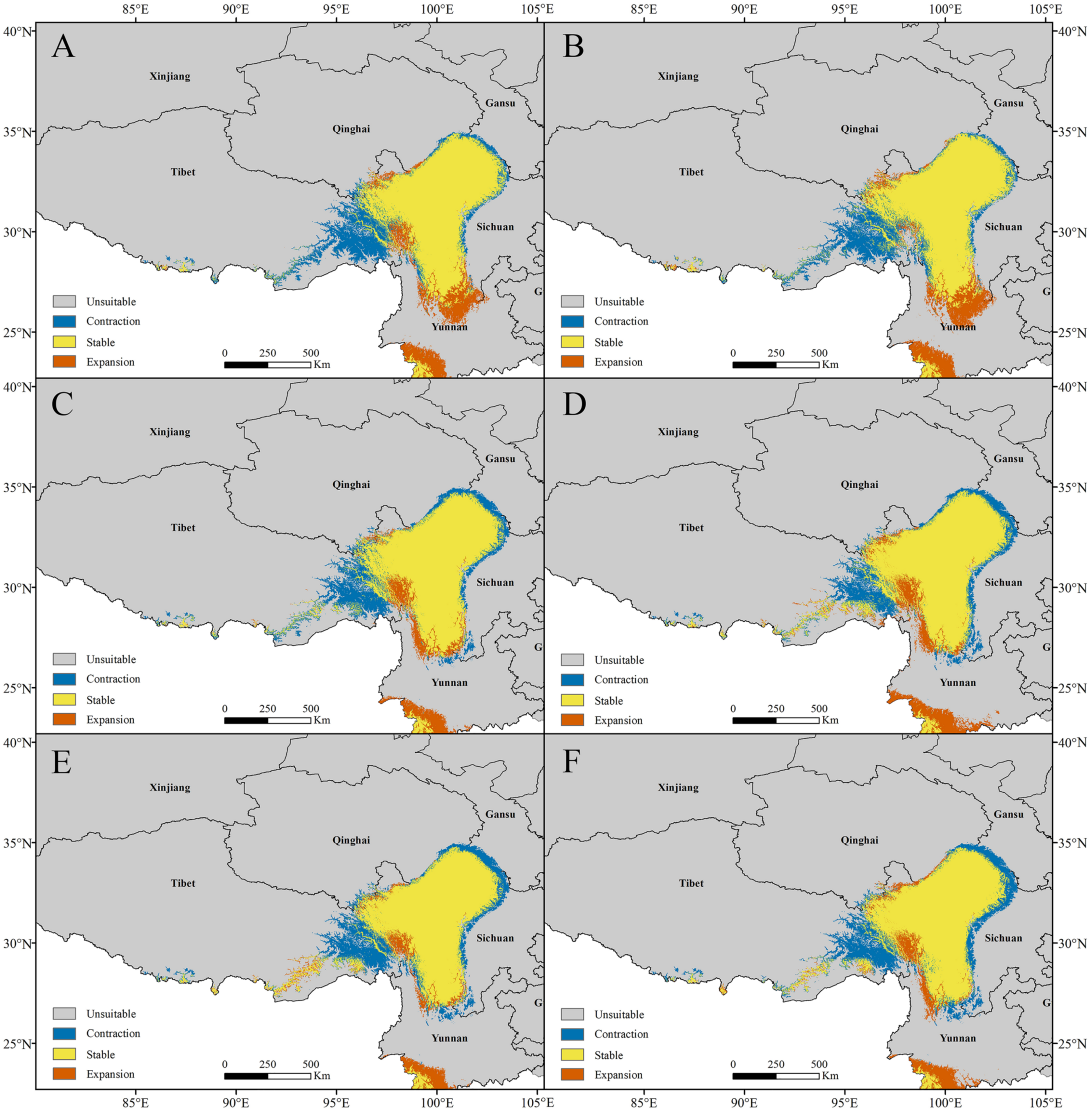
Changes in the potential distribution of *Pedicularis longiflora* var. *tubiformis* (Klotzsch) Tsoong under climate change scenarios. (A) RCP2.6-2050; (B) RCP2.6-2070; (C) RCP6.0-2050; (D) RCP6.0-2070; (E) RCP8.5-2050; (F) RCP8.5-2070.

In the RCP2.6 scenarios, the model prediction showed that 18.64% of the highly suitable area would be lost in 2050 and that 17.68% would be lost in 2070. In the RCP6.0 scenarios, the model prediction showed 18.42% of the highly suitable area would be lost in 2050 and that 16.98% would be lost in 2070. In the RCP8.5 scenarios, the model prediction showed that 19.69% of the highly suitable area would be lost in 2050 and that 20.81% would be lost in 2070 (Fig. 8). In general, the highly suitable area of the future potential distribution regions would be decreased by 0.62% in 2050 and 1.16% in 2070 under the RCP2.6 scenarios, by 3.46% in 2050 and 1.31% in 2070 under the RCP6.0 scenarios, and by 6.88% in 2050 and 5.49% in 2070 under the RCP8.5 scenarios.

In summary, the changes in the habitat areas of *P. longiflora* Rudolph and *P. longiflora* var. *tubiformis* (Klotzsch) Tsoong were slightly different under different scenarios and during different time periods. Although the highly suitable habitats of these two plant species decreased under the future climate scenarios, the total highly suitable habitat area was still very small.

### The change trend in high suitable habitat centroids

**Figure 9:**
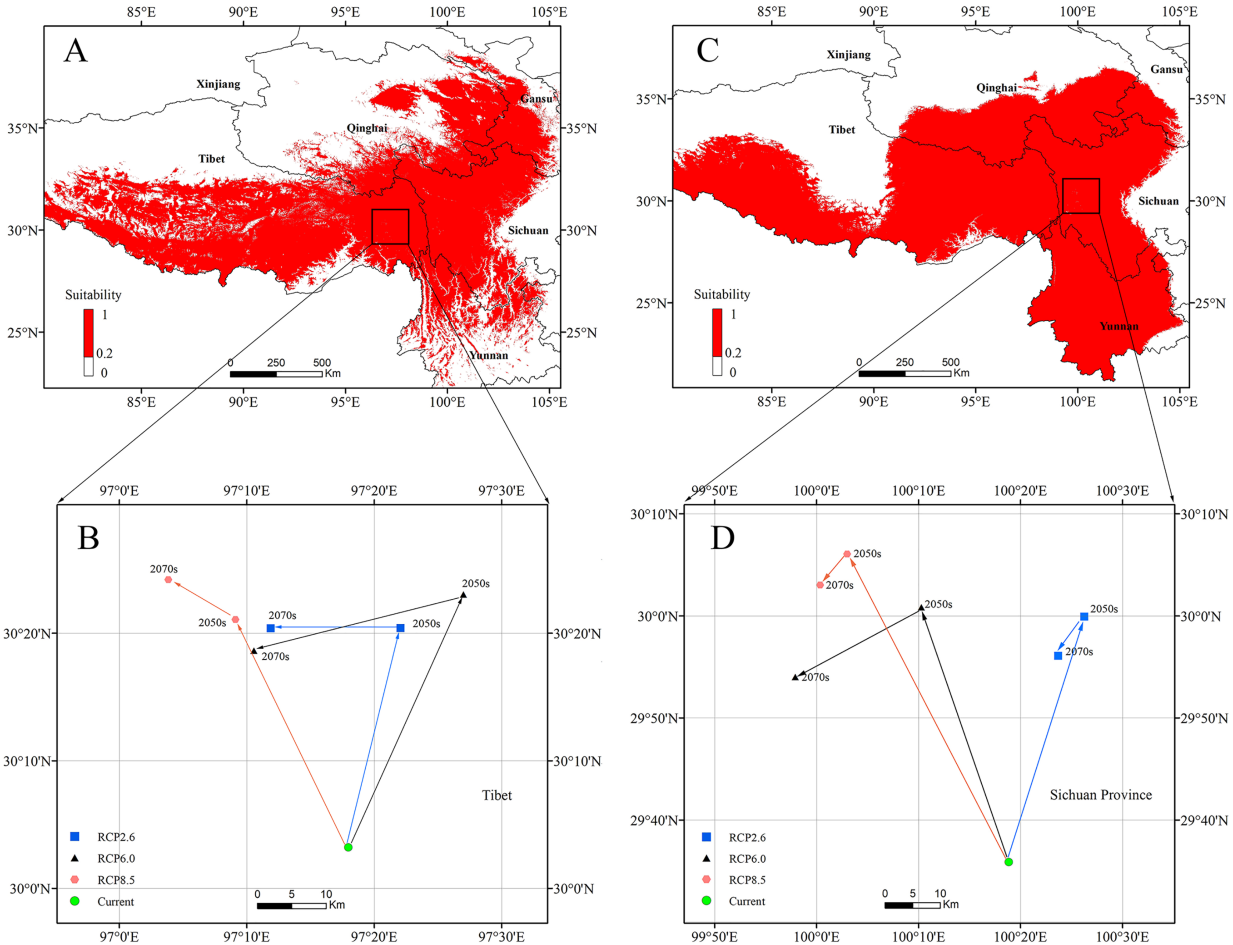
The current potential distributions (binary maps) (A), (C) and distributional shifts in highly suitable area centroids under climate change (B), (D). (A) and (B) *Pedicularis longiflora* Rudolph; (C) and (D) *Pedicularis longiflora* var. *tubiformis* (Klotzsch) Tsoong.

The centroids of the highly suitable region of *P. longiflora* Rudolph and *P. longiflora* var. *tubiformis* (Klotzsch) Tsoong are shown in Fig. 9. The centroid of *P. longiflora* Rudolph was located at 97°17′58″E and 30°03′12″N in Tibet under the current climate scenario. In the RCP2.6 scenarios, the centroid in 2050 was located at 97°22′03″E and 30°20′24″N, and the centroid in 2070 was located at 97°11′52″E and 30°20′23″N. The centroid shifted first to the northeast and then to the west in the RCP2.6 scenarios. In the RCP6.0 scenarios, the centroid in 2050 was located at 97°27′00″E and 30°23′02″N, and the centroid in 2070 was located at 97°10′34″E and 30°18′38″N. The centroid shifted first to the northeast and then to the southwest in the RCP6.0 scenarios. In the RCP8.5 scenarios, the centroid in 2050 was located at 97°09′07″E and 30°21′05″N, and the centroid in 2070 was located at 97°03′51″E and 30°24′12″N. The centroid shifted first to the northwest and then to the northwest in the RCP8.5 scenarios. In summary, the centroid of the highly suitable region for *P. longiflora* Rudolph was predicted to move northward (high latitude) from the present to the future.

The centroid of the highly suitable region for *P. longiflora* var. *tubiformis* (Klotzsch) Tsoong was located at 100°18′50″E and 29°35′52″N in Sichuan under the current climate scenario. In the RCP2.6 scenarios, the centroid in 2050 was located at 100°26′15″E and 29°59′56″N, and the centroid in 2070 was located at 100°23′42″E and 29°56′05″N. The centroid shifted first to the northeast and then to the southwest in the RCP2.6 scenarios. In the RCP6.0 scenarios, the centroid in 2050 was located at 100°10′17″E and 30°00′51″N, and the centroid in 2070 was located at 99°57′55″E and 29°54′00″N. The centroid shifted first to the northwest and then to the southwest in the RCP6.0 scenarios. In the RCP8.5 scenarios, the centroid in 2050 was located at 100°03′00″E and 30°06′05″N, and the centroid in 2070 was located at 100°00′21″E and 30°03′03″N. The centroid shifted first to the northwest and then to the southwest in the RCP8.5 scenarios. In summary, the centroid of the highly suitable region of *P. longiflora* var. *tubiformis* (Klotzsch) Tsoong was predicted to move northward (high latitude) from the present to the future.

## Discussion

The MAXENT model quantifies statistical relationships between predictor variables at locations where a species has been observed *vs* background locations in the study region. These relationships are constrained by various transformations of the original predictor variables (FCs) – allowing more FCs enables more flexible and complex fitting to the observed data. However, greater flexibility can increase the tendency of the model to overfit (Peterson et al., 2011). By default, the MAXENT model determines which FCs are allowed based on the number of occurrence positions in the dataset. Regardless of which FCs are allowed to be used in the model run, the MAXENT model provides protection against overfitting through regularization (Merow, Smith & Silander, 2013). The user can specify which FCs are allowed and adjust the regularization level through a single RM (default value = 1.0). However, few users set these parameters in practice, probably because doing so is laborious and time-consuming. Therefore, most empirical studies rely on the default settings of a given algorithm/package and potentially biased evaluation methods (Phillips & Dudík, 2008). In view of the above reasons, we suggest that the impact of the parameter settings of regularization and FCs on model structure and performance should be evaluated.

In this study, we used the information criterion method when comparing the adjusted model with the default model. Existing studies have shown that the most commonly used model quality indicator (AUC_TEST_) depends on the ability to predict the distribution of independent occurrences in the training area. This indicator is useful and intuitive, but it may lead to a level of confidence in the underlying model that may be unjustified (Godsoe, 2009). The average performance of the information criterion (AICc) (Akaike, 1973) is slightly greater than that of AUC_TEST_ and AUC_DIFF_ in larger datasets, but the difference is much greater when fewer data points are available, suggesting that the model selection approach based on the information criterion may be particularly useful when sample sizes are small. In addition, the amount of variation in the performance of the information criterion methods is much smaller than that in the AUC-based methods, which indicates that AICc selects the worst model from each analysis less frequently than the AUC-based method (Warren & Seifert, 2011). In view of the above reasons, we recommend using MAXENT’s AICc as an alternative heuristic method for the more commonly used AUC training and AUC testing.

The results of the MAXENT model showed that annual mean temperature, mean diurnal range, temperature seasonality, annual precipitation and annual mean temperature, mean diurnal range, isothermality, and annual precipitation were important factors affecting the potential distributions of *P. longiflora* Rudolph and *P. longiflora* var. *tubiformis* (Klotzsch) Tsoong and that these four important factors were climate factors. Three of them were temperature factors, indicating that temperature had a greater impact on the potential distribution of *P. longiflora* Rudolph and its variant. This result was consistent with previous studies (Ding, 2007). Similar to studies on the distribution of other plants on the Qinghai-Tibet Plateau (Cao et al., 2021; Han et al., 2021), we also found that temperature and precipitation factors are important factors affecting the potential distributions of *P. longiflora* Rudolph and *P. longiflora* var. *tubiformis* (Klotzsch) Tsoong. In the alpine meadow grassland ecosystem, precipitation is an important factor affecting plant growth (Bai, Hu & Zhao, 2021), precipitation mainly affects surface temperature through the feedback mechanism of soil moisture, air temperature is a direct factor that affects surface temperature, and surface temperature affects alpine the growth and development of alpine meadow plants (Zhou et al., 2015; Zhang et al., 2021) and then affects the distribution of plants. These climatic conditions restrict *P. longiflora* Rudolph and its variant to the high-altitude regions of the Himalayas and Hengduan Mountains. However, the Himalayas and Asian highlands, especially the “Third Pole”, are highly vulnerable to climate change and are the regions with the greatest sensitivity to and the most representative of global warming (Wei et al., 2021). Therefore, the impact of global warming on the potential distribution of *P. longiflora* Rudolph and its variant (*P. longiflora* var. *tubiformis* (Klotzsch) Tsoong) should not be underestimated.

This study showed that the potential distribution regions of *P. longiflora* Rudolph and *P. longiflora* var. *tubiformis* (Klotzsch) Tsoong would be reduced under different climate change scenarios (RCPs 2.6, 6.0 and 8.5) for 2050 and 2070. This finding was consistent with many studies on the impact of climate change on changes in the geographic distribution of several medicinal and threatened plant species distinguished by range contraction (Kumar, Singh & Sharma, 2019; Nagahama & Bonino, 2020; Rajpoot et al., 2020; Kumar, Rawat & Joshi, 2021). Existing studies have conducted climate change analysis with CCSM4, and the results have shown that despite the considerable uncertainties in these scenarios, the simulated climatic conditions in 2050 and 2070 indicated that the entire Qinghai-Tibet Plateau will experience significant warming in the coming decades. Considering the trend in future precipitation, it can be predicted that the Qinghai-Tibet Plateau will become warmer and drier in the future (Wei et al., 2021). However, the growth of *P. longiflora* Rudolph and *P. longiflora* var. *tubiformis* (Klotzsch) Tsoong required a cold and humid environment (Ding, 2007). Therefore, a warm and dry climate would aggravate the deterioration of the habitat and increase the survival risk. This will be a massive threat to *P. longiflora* Rudolph and its variants in the coming decades. Many studies have shown that global warming is a considerable challenge for alpine species. It is one of the main reasons for species redistribution, suitable habitat loss and species extinction (Ahmadi et al., 2019; Wang et al., 2019). To adapt to a warming environment, there is an interesting consensus that species ranges are always moving very high or higher (Hughes, 2011; Diez et al., 2020). Therefore, high-altitude mountain areas have always been a refuge for species sensitive to global warming (Harley & Paine, 2009; Diez et al., 2020). Although the strategy for adapting to global warming is to move to higher altitudes, if the altitude is very limited, the species’ immigration space will be very narrow. In future climate change scenarios, this will be a major challenge for species that prefer cold climates. Topographic relief also limits the ability of many alpine species to move to higher altitudes as temperatures increase (Hughes, 2011). The abovementioned reasons may be the main reasons that the suitable habitat area of *P. longiflora* Rudolph and its variants were predicted to shrink under future climate scenarios.

Along with climate change, increasing and irrational harvesting is attributed to escalating market demand for herbal medicine, and a lack of knowledge about sustainable harvesting leads to habitat degradation. Both the distribution of species and suitable habitat area would face a high risk of habitat loss in response to global climate change (Gajurel et al., 2014; Ranjitkar et al., 2016) and increasing biotic interactions among plant species at higher elevations (Liang et al., 2016; Kunwar et al., 2021). Hence, forecasting the climatic space of medicinal plants through a species distribution model can be crucial for habitat conservation and sustainable management of species in the future (Porfirio et al., 2014; Rana et al., 2020). The existing research indicates that many alpine medicinal plants are threatened by climate change and human factors (Applequist et al., 2019; Kumar, Singh & Sharma, 2019; Rana et al., 2020), and our research showed that climate change would adversely affect their sustainable use. Their habitats, the mountain areas of Southwest China, the Qinghai-Tibet Plateau, and the Himalayas, are likely to experience unpredictable alterations with climate change. These alterations would lead to the potential loss or shortages of local medicinal plant resources (Kumar, Singh & Sharma, 2019; Rana et al., 2020). In addition, overharvesting and intensive exploitation have also caused the species distribution and suitable habitat area of some other alpine medicinal plants to shrink (Wei et al., 2021). To solve these problems and address the challenges of climate change, a sustainable management plan should be developed for *P. longiflora* Rudolph and *P. longiflora* var. *tubiformis* (Klotzsch) Tsoong and also for other alpine medicinal species. Commercial cultivation is an effective strategy to address the disparity between the supply and demand of medicinal plants (Cheng et al., 2019; Wang et al., 2020). Our habitat suitability assessment method provided valuable information for developing planting plans, and the model determined where medicinal plants might be planted. With the above foundation, we wish to make the following suggestions: The first is to conduct surveys, collection and diversity evaluation of wild medicinal plant resources, which will help protect and domesticate species; the second is to strengthen the protection of the Qinghai-Tibet Plateau and the Himalayan-Hengduan Mountains. The ecological environment of these places was originally fragile and helped raise awareness of local medicinal plant protection and prevent unsustainable land use and excessive logging. The third suggestion is to build a commercial planting model. It is best to build a medicinal plant cultivation model that integrates mountain ecology, economic benefits and social benefits (Cheng et al., 2019; Shen, Yu & Wang, 2021). For example, an integrated management system of mixed cultivation for medicinal plants was proposed by Rana et al. (2020). Currently, only a few published articles have presented a systematic and comprehensive evaluation of mixed cultivation for medicinal plants in China (Chu et al., 2016; Moreira et al., 2018; Wu et al., 2019). Therefore, we need to further improve our understanding of the interactions between crops and medicinal plants and of how mixed cultivation determines the quality of herbs to achieve a balance between ecological, economic, and social gains in mountain areas.

## Conclusions

*P. longiflora* Rudolph and its variant (*P. longiflora* var. *tubiformis* (Klotzsch) Tsoong) are alpine plants and traditional Chinese medicines with important medicinal value, and future climate changes may have an adverse impact on their geographic distribution. Therefore, we used ENMeval to improve the MAXENT niche model and conducted an in-depth study on the potential distributions of *P. longiflora* Rudolph and *P. longiflora* var. *tubiformis* (Klotzsch) Tsoong. We adjusted the parameters of FC and RM in the MAXENT model, evaluated the adjusted MAXENT model using six indicators, determined the most important EGFs that affect the potential distributions of these plants, and compared their current potential distributions between the adjusted model and the default model. The adjusted model performed better; thus, we used the improved MAXENT model to predict the future potential distributions of these plants. *P. longiflora* Rudolph and its variant (*P. longiflora* var. *tubiformis* (Klotzsch) Tsoong) were predicted to move northward and showed a decreasing trend under future climate scenarios. This result is important for predicting their potential distribution regions under changing climate scenarios to develop effective long-term resource conservation and management plans for these species.

## Supplemental Information

10.7717/peerj.13337/supp-1 Supplemental Information 1 List of 22 Ecogeographical factors (EGFs).

10.7717/peerj.13337/supp-2 Supplemental Information 2 Species distribution occurrence records of *Pedicularis longiflora* Rudolph in China.

10.7717/peerj.13337/supp-3 Supplemental Information 3 Species distribution occurrence records of *Pedicularis longiflora* var. *tubiformis* (Klotzsch) Tsoong in China.

## Additional Information and Declarations

### Data Availability

The following information was supplied regarding data availability: The raw data are available in the Supplemental Files.
