# Supplementary material for: Feature tuning improves MAXENT predictions of the potential distribution of Pedicularis longiflora Rudolph and its variant
Source: PeerJ. 2022 May 3;10:e13337. doi: 10.7717/peerj.13337 (PMC9074863; doi:10.7717/peerj.13337)
Supplement: Supplemental Information 1 [file peerj-10-13337-s001.docx]

| Type | Ecogeographical Factors | Units |
| --- | --- | --- |
| Topo-graphical | Altitude | m |
|  | Aspect | ° |
|  | Slope | ° |
| Bio-climatic | Annual Mean Temperature | °C |
|  | Mean Diurnal Range (Mean of monthly (max temp−min temp)) | °C |
|  | Isothermality (Mean Diurnal Range/Temperature Annual Range) (*100) | - |
|  | Temperature Seasonality (standard deviation *100) | C of V |
|  | Max Temperature of Warmest Month | °C |
|  | Min Temperature of Coldest Month | °C |
|  | Temperature Annual Range (Max Temperature of Warmest Month−Min Temperature of Coldest Month) | °C |
|  | Mean Temperature of Wettest Quarter | °C |
|  | Mean Temperature of Driest Quarter | °C |
|  | Mean Temperature of Warmest Quarter | °C |
|  | Mean Temperature of Coldest Quarter | °C |
|  | Annual Precipitation | mm |
|  | Precipitation of Wettest Month | mm |
|  | Precipitation of Driest Month | mm |
|  | Precipitation Seasonality (Coefficient of Variation) | C of V |
|  | Precipitation of Wettest Quarter | mm |
|  | Precipitation of Driest Quarter | mm |
|  | Precipitation of Warmest Quarter | mm |
|  | Precipitation of Coldest Quarter | mm |
